# Dopaminergic regulation of hippocampal plasticity, learning, and memory

**DOI:** 10.3389/fnbeh.2022.1092420

**Published:** 2023-01-27

**Authors:** Theodoros Tsetsenis, John I. Broussard, John A. Dani

**Affiliations:** ^1^Department of Neuroscience, Mahoney Institute for Neurosciences, Perelman School of Medicine, University of Pennsylvania, Philadelphia, PA, United States; ^2^Department of Neurobiology and Anatomy, UT Health Houston McGovern Medical School, Houston, TX, United States

**Keywords:** ventral tegmental area, locus coeruleus, CA1, STDP, neuroanatomy

## Abstract

The hippocampus is responsible for encoding behavioral episodes into short-term and long-term memory. The circuits that mediate these processes are subject to neuromodulation, which involves regulation of synaptic plasticity and local neuronal excitability. In this review, we present evidence to demonstrate the influence of dopaminergic neuromodulation on hippocampus-dependent memory, and we address the controversy surrounding the source of dopamine innervation. First, we summarize historical and recent retrograde and anterograde anatomical tracing studies of direct dopaminergic projections from the ventral tegmental area and discuss dopamine release from the adrenergic *locus coeruleus*. Then, we present evidence of dopaminergic modulation of synaptic plasticity in the hippocampus. Plasticity mechanisms are examined in brain slices and in recordings from *in vivo* neuronal populations in freely moving rodents. Finally, we review pharmacological, genetic, and circuitry research that demonstrates the importance of dopamine release for learning and memory tasks while dissociating anatomically distinct populations of direct dopaminergic inputs.

## Introduction

As an organism navigates through an environment, its ability to associate relevance to specific contexts is crucial for survival. Mammals have evolved a system to form a cognitive spatial map of the environment relying on the hippocampus, a limbic structure required for the formation of new episodic memories (Smith and Bulkin, 2014). The hippocampus consists of excitatory neurons forming a trisynaptic loop, with local and hilar interneurons providing feedforward and feedback inhibition (Amaral and Lavenex, 2006). In addition, neuromodulation from two main sources influences hippocampal processing: the medial septal cholinergic and GABAergic projections that influence hippocampal theta rhythms (Bland and Oddie, 2001), and catecholaminergic projections from the midbrain and the *locus coeruleus* (LC; Madison and Nicoll, 1982; Otmakhova and Lisman, 1999). The association of events to contexts is facilitated *via* plasticity of synaptic connections, as first proposed by Hebb (1949). These synaptic changes are bidirectional and depend upon specific neural activity and protein synthesis (Abel et al., 1997). Importantly, not all events and contexts have similar relevance, and as such, neuromodulators such as catecholamines can enhance synaptic plasticity associated with an event, linking the synaptic change to the memory of the event (Frey and Frey, 2008; Shohamy and Adcock, 2010; Lisman et al., 2011).

Experimental evidence has shown that, as with other brain structures, changes in hippocampal synaptic plasticity and hippocampus-dependent learning are influenced by dopaminergic neurotransmission at these synapses. There are two main sources of dopamine (DA), one arising from the LC, and the other from the ventral tegmental area/*substantia nigra pars compacta* (VTA/SNc) of the midbrain (Duszkiewicz et al., 2019). The primary neurotransmitter of the LC, however, is norepinephrine (NE), which is released at a 10-fold concentration to that of DA (Kempadoo et al., 2016). By comparison, the VTA and midbrain DA projections have been shown with previous methods to have very sparse innervation of the hippocampus (McNamara et al., 2014; Kempadoo et al., 2016; Takeuchi et al., 2016). A confounding factor is that the hippocampus does not show significant expression of the DA transporter, which is the crucial anatomical marker of DA projections in other brain regions (Borgkvist et al., 2012). A goal of this review is to provide the most recent evidence in support of a direct anatomical projection from dopaminergic VTA neurons to the hippocampus. Then, DA neuromodulation of hippocampal synapses will be discussed within the framework of spike timing dependent plasticity (STDP). Finally, the relevance of dopaminergic neurotransmission to learning and memory will be reviewed.

## Hippocampal innervation from midbrain dopaminergic sources

The existence of direct dopaminergic innervation in the rodent hippocampus has been historically controversial, with this controversy persisting until recently. The low extracellular concentrations of DA in the hippocampal formation, especially in comparison to the striatum, have initially led scientists to believe that the hippocampus was completely devoid of DA innervation (Lindvall and Bjorklund, 1978).

The development and use of anterograde and retrograde fluorescent tracing techniques provided the first strong indications of midbrain dopaminergic innervation of the hippocampus (Gasbarri et al., 1997). The first evidence came from experiments using co-localization analysis of retrogradely transported True Blue (TB) from the hippocampus with tyrosine hydroxylase (TH) expressing cells in midbrain dopaminergic areas (Swanson, 1982). It was shown that following TB injection in different rat hippocampal regions [CA1, CA3, and dentate gyrus (DG)], retrogradely-labeled DA neurons could be identified in the rostral VTA and SNc. These results found further support from studies analyzing TH-positive fibers in the hippocampus of NE-depleted rats (Verney et al., 1985) that demonstrated the existence of DA innervation predominantly in the CA1 and subiculum and to a lesser extent in the CA3 and DG.

Electrolytic and chemical lesions of the VTA/SNc or their hippocampal terminals also indicated direct midbrain dopaminergic innervation of the hippocampus. In these lesion experiments, rats were pretreated with hippocampal injections of dismethylimipramine to prevent degeneration of noradrenergic terminals and then given an injection of the neurotoxicant 6-hydroxydopamine (6-OHDA) into the dorsal hippocampus. This selective monoaminergic toxin caused destruction of midbrain DA afferents, producing a dramatic decrease in DA content in the dorsal hippocampus (Itoh et al., 1984). Moreover, chemical and electrolytic lesions of the VTA and SNc (Scatton et al., 1980) led to a severe reduction of hippocampal DA and 3,4-Dihydroxyphenylacetic acid (DOPAC) concentrations. Taken together, these data suggest that an important portion of hippocampal afferents are dopaminergic and originate from the VTA/SNc regions.

Almost a decade later, studies by Gasbarri et al. (1994a,b, 1996) provided a rigorous analysis of the topographical distribution of midbrain DA neurons and their projections to the different regions of the hippocampal formation of the rat. Their data showed a direct innervation of the hippocampus from the VTA, SNc, and retrorubral field (RRF) that was more prominent at CA1 and subiculum in the ventral part of the hippocampus as shown previously (Verney et al., 1985). By combining retrograde tracing using Fluoro Gold (FG) and TH immunostaining, they quantified that only a part (15%–18%) of this innervation is actually dopaminergic. Importantly, these dopaminergic projections did not produce any collaterals to other regions.

Despite all this evidence, the extent of hippocampal innervation by midbrain dopaminergic sources in the rat brain remained underwhelming. Techniques using genetic mouse models and cell-type specific viral tracing approaches allowed more recent studies to analyze this innervation in the mouse brain. These endeavors were mainly hindered by the inefficiency of retrograde transport from dopaminergic terminals (Tervo et al., 2016). Some of these studies have attempted to map midbrain dopaminergic projections to the dorsal CA1 (dCA1) hippocampal field using axon-targeted channelrhodopsin (ChR2) viral methodologies (McNamara et al., 2014; Kempadoo et al., 2016; Takeuchi et al., 2016). All of these studies have provided similar results that showed sparse innervation with only a limited number of dopaminergic fibers present in the dorsal hippocampus and the dCA1 region in particular. Since ChR2 is not an anterograde tracer, it is likely that these results underestimated the amount of hippocampal innervation that stems from midbrain dopaminergic sources. In fact, several large-scale input-output tracing efforts (Beier et al., 2015; Lerner et al., 2015; Poulin et al., 2018) have omitted the hippocampus from their analysis mainly due to the sparse dopaminergic innervation in this area. These discrepancies suggested the existence of alternative sources of DA in the rodent hippocampus, namely the classic NE area, the LC (Devoto et al., 2001; Smith and Greene, 2012). LC neurons synthesize DA as a precursor of NE, and they have been shown to co-release both neurotransmitters in the hippocampus, acting as an alternative source of DA (Smith and Greene, 2012; Kempadoo et al., 2016; Takeuchi et al., 2016). These data refueled the controversy regarding the source of DA in the rodent hippocampus and created a narrative that all physiologically relevant DA functionality comes almost entirely from the LC.

**Figure 1:**
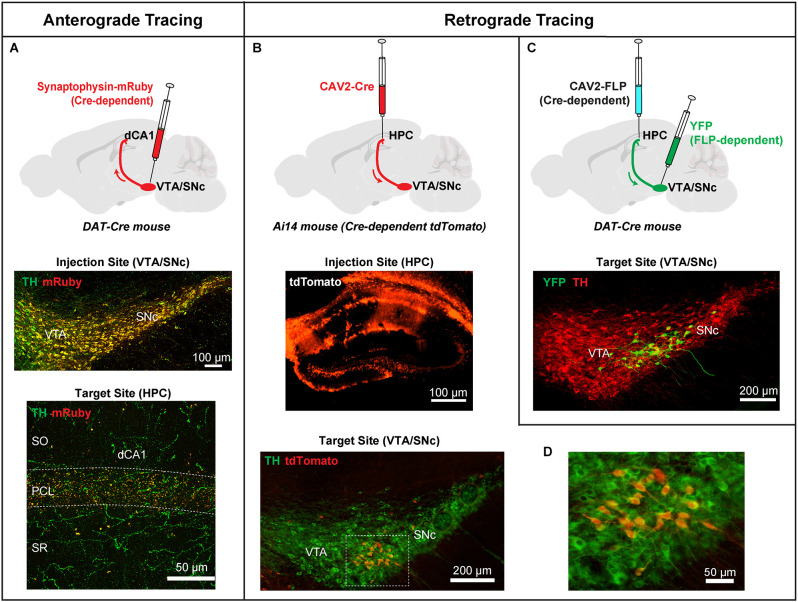
Anterograde and retrograde viral tracing approaches to label direct projections from midbrain dopaminergic neurons to the dorsal hippocampus (HPC). **(A)** Top: An AAV expressing a Cre-dependent Synaptophysin-mRuby fusion protein was injected into the VTA/SNc of DAT-Cre mice to facilitate anterograde labeling of DA terminals originating from the midbrain. Middle: Coronal section of the site of injection in the VTA/SNc showing infection with Synaptophysin-mRuby (red), and DA cells labeled with an antibody against TH (green). Synaptophysin-mRuby infection is restricted to DA cells in this region indicated by the colocalization with TH (orange/yellow). Bottom: Coronal section of dCA1 showing punctate DA terminals expressing Synaptophysin-mRuby and TH (orange/yellow) demonstrating the extent of DA innervation from VTA/SNc. **(B)** Top: A CAV2 expressing Cre was injected in the dorsal HPC of Ai14 mice. This procedure expresses Cre in all neurons projecting to the hippocampus with induction of tdTomato expression. Middle: Coronal section showing infection of the dorsal HPC of an Ai14 mouse injected with CAV2-Cre and the induction of tdTomato expression in infected cells. Bottom: Image from the VTA/ SNc of an Ai14 mouse infected with CAV2-Cre in the hippocampus and co-labeled for TH (green). **(C)** Top: A CAV2 expressing a Cre-dependent version of FLP recombinase was injected in the HPC of DAT-CRE mice, producing FLP expression only in DA cells that project to the hippocampus. An AAV expressing a FLP-dependent version of YFP was injected in the VTA/SNc region of the same mice. DA cells projecting to the hippocampus are identified by YFP expression. Bottom: DA neurons (green) located at the dorsolateral VTA/SNc project directly to the hippocampus. Note the similarity between bottom panels in **(B)** and **(C)**. **(D)** Higher magnification of the box in bottom **(B)** showing TH-positive cells co-expressing tdTomato (red), identifying putative DA neurons that project to the hippocampus. Adapted from Tsetsenis et al. (2021).

To address the source of DA innervation, two recent studies used a sensitive anterograde viral tracing methodology to examine the distribution of direct midbrain dopaminergic projections to the dCA1 (Broussard et al., 2016; Tsetsenis et al., 2021). This method utilizes an adeno-associated virus (AAV) to express the synaptic vesicle protein synaptophysin fused with a fluorescent protein exclusively in midbrain dopaminergic neurons of the VTA/SNc (Figure 1A). Because synaptophysin is expressed multiple times in the many synaptic vesicles in each axonal terminal, the localization of synaptophysin fusion proteins results in an enriched fluorescent signal in DA neuronal terminals enhancing the sensitivity of detection (Wiedenmann and Franke, 1985). Using synaptophysin fluorescence, prominent punctate fluorescent terminals were identified along the pyramidal cell layer (PCL) in the dCA1 (Broussard et al., 2016; Tsetsenis et al., 2021). Additionally, sparse fiber tracts were observed in *stratum oriens* (SO) and *stratum radiatum* (SR) in agreement with what was previously shown with ChR2 expression. These labeled puncta and fibers were also immuno-positive for TH, confirming their midbrain dopaminergic identity.

To differentiate the VTA/SNc and LC sources further, Tsetsenis et al. (2021) generated conditional knockout mice in which TH was specifically ablated from all neurons expressing DA beta-hydroxylase (Dbh), which catalyzes the conversion of DA to NE. Thus, in these mice, TH is removed from noradrenergic neurons in the LC (TH^LC^ KO), which is the source of noradrenergic input to the dorsal hippocampus. The procedure leaves midbrain dopaminergic fibers and terminals as the sole source of the TH signal in the hippocampus. TH immunofluorescence was significantly reduced in the dCA1 region of TH^LC^ KO mice, particularly in the SO and SR, but a substantial signal remained in the PCL. The remaining TH signal accounted for one-third of the TH signal of control mice. This smaller signal strongly resembled the VTA/SNc innervation to the dCA1 seen using the synaptophysin anterograde tracing approach, indicating that it represents the midbrain dopaminergic input.

To identify the midbrain cell population that directly projects to the dorsal hippocampus, the same study used two viral retrograde strategies (Figures 1B–D) based on canine adenovirus type 2 (CAV2; Soudais et al., 2001) in combination with TH immunohistochemistry and cell-type specific viral and genetic expression of fluorescent markers (Tsetsenis et al., 2021). Injection in the dorsal hippocampus with these retrograde viruses labeled a population of DA neurons near the interface of SNc and lateral VTA. These cells exhibited electrophysiological properties (i.e., firing frequency, I_h_ current amplitude, input resistance, resting membrane potential) typical of dopaminergic neurons (Tsetsenis et al., 2021). These findings provide strong evidence for the existence of a direct DA innervation in the hippocampus originating from midbrain dopaminergic sources and add important information to the topographical chart of VTA/SNc connectivity, with a focus on a target region that has often been overlooked in DA circuit-mapping analyses. Next, we will review how these two distinct DA sources modulate hippocampal plasticity and learning and memory processes.

## One mechanism of dopaminergic modulation of hippocampal plasticity

Given that anatomical evidence indicates the presence of D1 and D5 receptors in the hippocampus (Savasta et al., 1986; Wamsley et al., 1992) and the anatomical tracing studies highlighted in the previous section, how does dopaminergic transmission in the hippocampus influence information processing in the hippocampus? Early studies of intact synapses from brain slices provided evidence that D1-type receptors facilitate long-term potentiation (LTP) in the CA1 *via* activation of protein kinase A (PKA; Huang et al., 1995; Kentros et al., 2004; Muzzio et al., 2009). Because D1/D5 receptors are positively coupled to cAMP-PKA, drugs that enhanced this pathway attenuated age-related deficits in memory and LTP (Bach et al., 1999). PKA was also shown to be involved in maintaining the stability of place fields in the CA1. Blockade of PKA had the same effect as blockade of protein synthesis: i.e., it impairs the long-term stability of hippocampal place fields (Rotenberg et al., 2000; Agnihotri et al., 2004; Kentros et al., 2004) and also the ability to retain long-term memories.

The most prominent model proposed to synthesize these findings is the synaptic tag and capture model by Frey and Morris (1997, 1998) and (Viola et al., 2014). This model posits that strong stimulus events induce a synaptic tag comprised of the phosphorylation of receptors or kinases. The second signal, coming from dopamine receptor activation, activates the cAMP/PKA pathway which synergistically act with the synaptic tag to produce plasticity related proteins (O’Carroll and Morris, 2004).

The synaptic tag and capture model has provided a framework to explain how Hebbian processes in the hippocampus can be modified by catecholamines (Lisman et al., 2011). Complementing this conceptual model is evidence that dopamine modifies spike timing dependent plasticity (STDP). STDP is a biologically relevant model for hippocampal plasticity that provides evidence for a Hebbian model of hippocampal circuitry underlying learning and memory. STDP requires the coordinated generation of postsynaptic action potentials and presynaptic stimulation within a limited temporal window (Bi and Poo, 1998, 1999, 2001; Tao et al., 2000). STDP is a refinement on Donald Hebb’s treatise that the correlated activity of hypothetical groups of neurons called “cell assemblies,” a central tenant of which is the importance of temporal order and the spatial specificity ensuring that only activated synapses become modified (Hebb, 1949; Bi and Poo, 2001). Early studies investigated the synaptic modifications of entorhinal input to the DG (Levy and Steward, 1983). Specifically, they tested associativity of the synaptic input to the DG and found that a weak stimulation preceding a strong stimulation by as much as 20 ms, would potentiate the response to the weak stimulation. However, if the strong stimulation preceded the weak stimulation, then the postsynaptic response to the weak stimulation would be depressed.

Postsynaptic spiking is an important event in the synaptic plasticity process (Nicoll and Malenka, 1995; Bi and Poo, 1998, 2001). Postsynaptic neurons fire action potentials, and the active properties of dendrites allow the spike initiating at the axon hillock to back-propagate into the dendrites. This backpropagation provides a precise timing signal to dendritic synapses to play an active role in associative synaptic modification. In the CA1, it was shown that back-propagating spikes coupled with subthreshold stimulation evoked a higher Ca^2+^ influx, potentiating the synapse (Magee and Johnston, 1997). Several channels contribute to the postsynaptic response, including NMDA channels and A-type potassium channels at the postsynaptic site.

**Figure 2:**
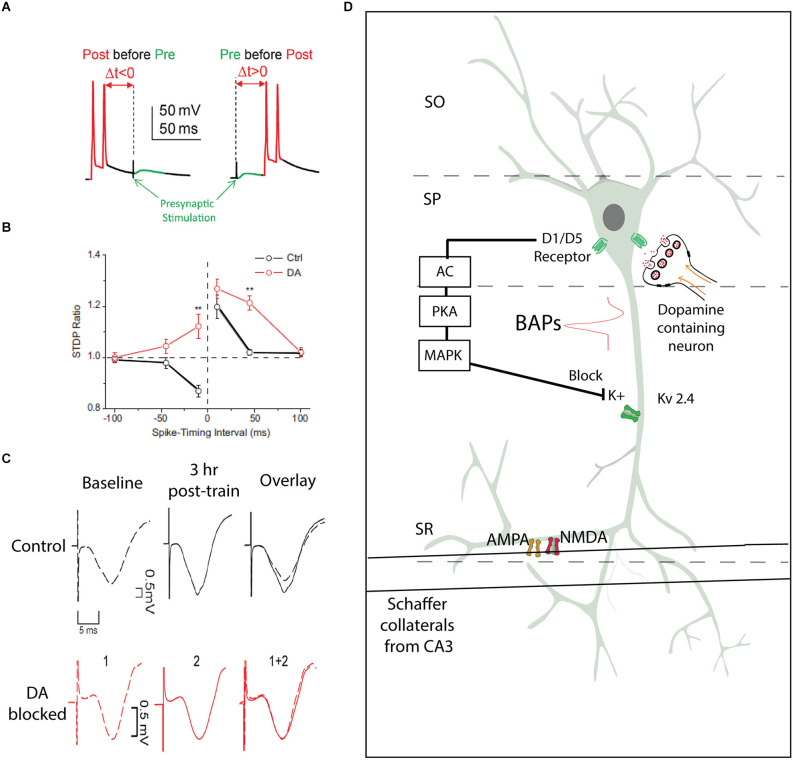
Spike timing dependent plasticity as a model for synaptic plasticity underlying hippocampal learning. **(A)** Experimental protocol from patch clamp experiments in cell-attached mode, two action potential produced on the recorded cell following by a presynaptic stimulation, or presynaptic stimulation preceding action potentials (adapted from Yang and Dani, 2014). **(B)** Illustration from Zhang et al. (2009) with black lines demonstrating responses of pyramidal neurons showing a characteristic STDP response, and the addition of extracellular dopamine broadening the temporal window and enabling greater sensitivity to presynaptic stimulation by CA1 pyramidal neurons. **P < 0.01, Student’s *t*-test. **(C)** An aversive learning event produces an increase in the fEPSP amplitude, an effect that is blocked with the administration of D1 antagonist SCH23390 (adapted from Broussard et al., 2016). **(D)** Model illustrating the role of dopamine in results from **(B)** and **(C)**. Input from the Schaffer Collaterals make synaptic connections with AMPA and NMDA receptors in the SR of a CA1 pyramidal neuron. If this presynaptic input follows an action potential, backpropagating actions potentials (BAPs) can cause voltage gated Kv2.4 channels to open, which serve to limit the influence of presynaptic input. Dopaminergic projections to the soma of CA1 pyramidal neurons can trigger a cascade that will activate MAPK, which block Kv2.4 channels, removing the inhibition of presynaptic input by postsynaptic action potentials. AC, Adenylyl cyclase; PKA, protein kinase A; MAPk, mitogen-activating protein kinase; BAPs, backpropagating action potentials; SR, stratum radiatum; SO, stratum oriens; SP, stratum pyramidale; Kv2.4, voltage gated potassium channel.

In a series of experiments recorded from the granule cells of the DG, the spike timing dependent protocol was demonstrated in an intact slice to produce LTP and long-term depression (LTD) in specific timing protocols (Yang and Dani, 2014). When D1 or D5 receptors were inhibited either pharmacologically or genetically (with KO mice), the same spike timing protocol failed to induce LTP. Furthermore, the addition of D1/D5 receptor agonist SKF81297 in intact mice would facilitate LTP while using an STDP protocol otherwise typically produces LTD. Mechanistically, this effect on postsynaptic plasticity was dependent upon potassium channels. Mice that lacked the inward rectifying Kv4.2 channel were less likely to show the effect of D1 receptor antagonists, and the use of selective I_A_ channel blocker 4-AP mimicked the faciliatory effect of D1 receptor agonists. D1 and D5 receptors signaling through adenylyl cyclase and PKA results in activation of mitogen-activated protein kinase (MEK). MEK in turn acts to inactivate *I_A_* channels (see Figure 2). Blocking *I_A_* channels serves to boost back-propagating action potentials from the pyramidal soma, which is the mechanism underlying the coincidence of presynaptic activity and postsynaptic response in the STDP protocol. Thus, in a biological system, dopamine serves to facilitate and widen the temporal window of STDP, which would influence information processing throughout the hippocampus. This evidence provides a model for a mechanism of dopamine modulation of Hebbian plasticity. These mechanistic experiments examined the perforant path to DG circuit, but dopamine had a similar effect on CA1 synapses, namely, an increase in the gain of sensitivity and a loss of temporal contrast of the spike timing mechanism (Zhang et al., 2009). In both the CA1 and the DG, dopaminergic modulation of spike timing dependence required active neurotransmission of NMDA receptors. Taken together, these findings provide evidence of a biological mechanism underlying dopaminergic modulation of synaptic plasticity (Figure 2).

## The relevance of dopaminergic modulation in learning and behavior

The dopaminergic modulation of STDP provides mechanistic insight into the channels and subunits underlying Hebbian synaptic plasticity, but assessment of measures of plasticity following hippocampus-dependent learning is required in order to provide evidence of the necessity of dopaminergic neurotransmission to learning. One common technique is to measure the AMPA/NMDA (A/N) ratio of hippocampal slices extracted* ex vivo* from mice that had recently undergone a learning procedure, such as the novel object recognition (NOR) task (Yang et al., 2017). In the NOR task, a mouse sees two objects in an open field, and then in the next trial one object is changed, the novel object. Mice spend more time exploring the novel object rather than the unchanged object. The A/N ratio is a common measure of synaptic plasticity, usually indicating an increase in specific postsynaptic AMPA receptors following a plasticity-inducing learning event. Previous evidence indicated that the DG was crucial in the detection of novelty (Rolls and Kesner, 2006; Kesner and Rolls, 2015), and thus whole cell patch clamp recordings from granule cells of DG neurons were used to measure changes in the A/N ratio of these neurons. While recording, stimulation was passed through the perforant pathway, which is the bundle of axons projecting from the entorhinal cortex to the DG. The NOR task provided evidence of an anatomical dissociation of hippocampal function. In the DG, NOR produces an increase in the A/N ratio, driven specifically by an increase in the AMPA current following the introduction of a novel object compared to mice that had seen identical object pairs (without novelty; Yang et al., 2017). By contrast, in the CA1, no novelty-related plasticity was observed.

In contrast, inhibitory avoidance (IA) footshock training in a distinct context produced plasticity in a different circuit of the hippocampus. In this task, mice are in a well-lighted space, and a door opens to a dark, unlit space. The mice quickly move to the dark space where they are given a footshock. Thus, in subsequent trials, the mice delay entering the dark space when the door opens. IA training produced an increase in the A/N ratio in the synapses arising from the Schaffer collateral/CA1 pathway, but synapses in the perforant pathway remain unchanged (Broussard et al., 2016). This functional dissociation remains consistent with the role of the DG in pattern separation in an environment (Rolls and Kesner, 2006), compared to the contextual discrimination that requires the CA1.

Dopaminergic neurotransmission was necessary for the changes in the A/N ratio observed in both the NOR and IA studies. When animals were injected with a D1-type receptor antagonist, SCH23380, during the learning of the novel-object task, the A/N ratio remained unchanged. These experiments were confirmed with D1- and D5-receptor KO mice, indicating a role for both receptors in novelty-related plasticity. For the IA task, there was an increase in the field excitatory postsynaptic potential (fEPSP) amplitude, and the hippocampal synaptic potentiation was contingent upon intact DAergic neurotransmission. Specifically, the administration of D1 receptor antagonists just prior to and during training blocked the acquisition of long-term memory and the changes in the fEPSP.

A second measure of plasticity, taken *in vivo* from freely moving animals, is the fEPSP. The fEPSP measures the postsynaptic responses of a neuronal population in the vicinity of the recording electrode (Clarke et al., 2010). Behavioral experiments can be paired with high or low-frequency stimuli, and it can be determined whether these behaviors contribute to long-term plasticity. For example, an exploration task bidirectionally enhanced synaptic plasticity. That is, if paired with a weak low-frequency stimulus, environment exploration produced LTD, and if paired with a high-frequency stimulation, exploration produced LTP in the CA1. This bidirectional plasticity was once again dependent upon D1/D5 receptor activity (Lemon and Manahan-Vaughan, 2006, 2012).

## How drugs of abuse hijack dopaminergic mechanisms that influence learning

Long-term potentiation is one mechanism that drugs of abuse utilize to hijack dopaminergic modulation of hippocampal function (Ungless et al., 2001). For example, an injection of biologically relevant doses of nicotine enhances the fEPSP in the DG (Tang and Dani, 2009; Zhang et al., 2010). Local infusions or systemic injections of D1 receptor antagonists into the hippocampus block drug-induced potentiation (Jenson et al., 2015). Other drugs of abuse such as cocaine and methamphetamine also increase plasticity in the hippocampus (Broussard et al., 2012; Jenson et al., 2015). When paired with a low amplitude theta burst (which does not by itself potentiate these synapses), a low dose of methylphenidate increases the population spike amplitude in the DG (Jenson et al., 2015). A low dose of methylphenidate has also been shown to extend the temporal window of STDP in the DG, in a dopamine-dependent manner. The dose that was shown to enhance plasticity also enhanced memory retention in a delayed non-match-to-position task.

## Dopaminergic modulation of hippocampus-dependent cognitive behaviors

### Pharmacological studies

A requirement for DA transmission in the hippocampus for mnemonic processing has been established *via* pharmacological manipulations that block or enhance DA receptor signaling during hippocampus-dependent learning and memory tasks. The first indication came from research using aged mice where systemic infusion of the partial D1/D5 receptor agonist SKF38393 improved spatial memory performance in the Barnes maze task (Bach et al., 1999). On the other hand, inhibition of D1-like receptor signaling by systemic injection of the D1/D5 receptor antagonist SCH23390 inhibited nicotine-induced conditioned place preference in mice (Tang and Dani, 2009) and caused an impairment in NOR (Yang et al., 2016). In these cases, the behaviors were associated with dopaminergic regulation of long-term plasticity in the hippocampal DG, and the plasticity was inhibited by local infusion of SCH23390 into the DG (Tang and Dani, 2009).

Strong evidence was also provided by other studies that used local pharmacological manipulations in the hippocampal region. Experiments testing working memory in the 8-arm radial maze have shown that rats can improve performance in specific tasks after post-training intra-hippocampal infusions of DA receptor agonists. Infusion of d-amphetamine and D1 and D2 receptor agonists in the dorsal hippocampus (Packard and White, 1991) facilitated win-shift retention, while it had no effect on win-stay acquisition. In a different study (Wilkerson and Levin, 1999), infusion in the ventral hippocampus of the D2 receptor agonist quinpirole resulted in a dose-dependent improvement in choice accuracy, while the same treatment with the D2 receptor antagonist raclopride caused deficits in this task. Local inhibition of D1 and D2 receptors in the intermediate CA1 was also shown to cause an impairment in the acquisition and reinstatement of morphine-induced conditioned place preference (Assar et al., 2016). These results are in agreement with studies that examined the distribution of D1 and D2 receptors in the hippocampus (Gangarossa et al., 2012; Puighermanal et al., 2015, 2017) demonstrating an increasing gradient of expression of D2 receptors along the dorso-ventral axis (Dubovyk and Manahan-Vaughan, 2019).

Dopaminergic signaling in the hippocampus has also been shown to be required and to modulate spatial learning and memory. In rats performing a delayed matching-to-place task in the Morris water maze, bilateral intrahippocampal infusion of the D1/D5 receptor antagonist SCH23390 during encoding caused an impairment in memory retrieval (O’Carroll et al., 2006). Similar results were obtained when SCH23390 was infused in the dCA1 immediately after each training session in the spatial memory version of the Morris water maze task, while delivery of SKF38393 in the same region significantly improved spatial memory retention (da Silva et al., 2012). It was also shown that spatial learning of the platform position increased DA levels in the DG of rats, a process that was impaired by blockade of D1/D5 receptor signaling locally in the DG (Wang et al., 2019). In the alternating T-maze task, bilateral infusion into the DG of mice of SKF81297 produced an enhancement in spatial memory (Kern et al., 2015). Pharmacological inhibition of D1-like receptor signaling in mouse hippocampal CA3 was also shown to cause an impairment in recognition memory of a novel context (Wagatsuma et al., 2018). Spatial object recognition memory was demonstrated to induce DA release in the dorsal hippocampus, and this type of memory was blocked by pharmacological lesion of catecholamine fibers in the dorsal hippocampus (Moreno-Castilla et al., 2017).

A role for DA in the regulation of hippocampus-dependent associative memory was first demonstrated in the step-down version of IA. Blocking D1/D5 receptor signaling by bilateral SCH23390 infusions into dCA1 either prior to testing (Barros et al., 2001) or at different time windows within 12 h after training (Bernabeu et al., 1997; Rossato et al., 2009) impaired recall suggesting an involvement of local DA signaling in memory retrieval. At the same time frame, activation of D1-type signaling by infusion of SKF38393 increased recall performance. However, a more recent study (Broussard et al., 2016) has demonstrated a requirement for DA in the dCA1 during memory acquisition during the light-dark version of IA. In this study, SCH23390 delivered either systemically or bilaterally in the dorsal hippocampus before training impaired memory recall the next day. Moreover, activation of D1-type receptor signaling in the dorsal hippocampus before training with SKF81297 enhanced memory retention 3 days after the initial training. In a different set of experiments (Menezes et al., 2015), novelty exposure after IA training increased hippocampal DA levels and facilitated extinction, a phenomenon that was inhibited by intrahippocampal infusion of SCH23390.

Hippocampal DA has also been shown in many studies to be an essential modulator of contextual fear conditioning (cFC), another hippocampus-dependent task of associative learning (Kim and Fanselow, 1992). In rats, subcutaneous injection of SCH23390 inhibited the acquisition of cFC, while delivery 30 min after encoding did not have an effect on recall (Inoue et al., 2000). Infusion of SCH23390 into the dorsal hippocampus before cFC decreased freezing during long-term memory testing the next day in rats (Heath et al., 2015) and mice (Tsetsenis et al., 2021; Sayegh et al., 2022). Similar results were obtained when SCH23390 was infused into the ventral hippocampus either 10 min before or 12–14 h after cFC training (Karunakaran et al., 2016). Activation of D1 receptor signaling by bilateral infusion of SKF81297 into the DG enhanced extinction of contextual fear in mice (Kern et al., 2015). New research also indicates that hippocampal DA is required for the acquisition of trace fear conditioning memory (Wilmot et al., 2022). Taken together, these pharmacological manipulations establish an essential role of DA signaling in the hippocampus for a variety of cognitive processes of learning and memory.

### Studies using DA receptor knockout mice

Further evidence for the role of hippocampal DA signaling in learning and memory comes from the behavioral characterization of mice lacking specific types of DA receptors. D1-receptor knockout mutant mice show deficits in spatial memory acquisition in the Morris water maze task (El-Ghundi et al., 1999; Granado et al., 2008; Xing et al., 2010), while mice lacking either D3 (Xing et al., 2010) or D5 receptors (Granado et al., 2008) exhibit normal learning abilities in this test. D1-receptor knockout mice also show spatial memory deficits in the Barnes maze, as well as in associative learning when subjected to tests of active avoidance, fear, and eyeblink conditioning (Ortiz et al., 2010). Interestingly, the same impairments were reproduced with hippocampus-specific knockdown of D1 receptors using small interfering RNA injections in the hippocampus (Ortiz et al., 2010). Additionally, conditional knockout of D1 receptors in the DG of mice was also shown to impair cFC recall, but the same genetic manipulation in D5 receptors had no effect on cFC memory (Sarinana et al., 2014). The consensus of these studies indicates D1 receptors to be the primary mediators of DA modulation of hippocampus-dependent memory processes.

Recordings from the CA1 of D1-receptor knockout mice show that place cells from these mice are impaired in remapping to manipulations to cues in the environment (Tran et al., 2008). Similar experiments in D2-receptor knockout mice show that place cells from these mice are generally impaired relative to place cells in WT mice. Specifically, D2 KO place cells were impaired in spatial tuning, had lower intra-field firing rates and were less stable than WT. Unlike place cells in D1 KO mice, D2 KO mice had similar rates of remapping to changes in proximal spatial cues (Nguyen et al., 2014).

### Circuit-specific studies

The pharmacological and genetic studies mentioned above have established the importance of DA signaling in the hippocampus for different forms of learning and memory. However, they cannot provide information about the origin of the dopaminergic signal and the sources that are activated during these cognitive processes. Although midbrain VTA/SNc dopaminergic centers have been considered the main DA input to the hippocampus, recent evidence implicates the LC as another source of DA neurotransmission in this region (Devoto et al., 2001; Smith and Greene, 2012). The development of circuit-specific methods to control neurotransmitter release using optogenetics (Tye and Deisseroth, 2012) has offered powerful tools to dissect the contribution of these two dopaminergic sources to hippocampus-dependent forms of memory.

The idea of dopaminergic contributions from the LC in hippocampus-dependent memory processes found support in a spatial learning task of episodic memory (Takeuchi et al., 2016). In this task, optogenetic activation of the LC shortly after encoding of a reward location in a familiar arena enhanced retention of the location memory 24 h later. This enhancement was blocked by intrahippocampal infusion of a D1/D5 receptor antagonist but not by a beta-adrenergic receptor antagonist indicating that the effect was modulated by DA. Similarly, optogenetic stimulation of LC terminals in the dorsal hippocampus during acquisition of a spatial object recognition task resulted in improved learning (Kempadoo et al., 2016). Again, these effects did not depend on beta-adrenergic receptor function but were abolished with pre-training inhibition of D1-like receptors in the dorsal hippocampus.

Both the above studies provided compelling evidence for a neuromodulatory role of hippocampal DA originating from the LC on the formation and retention of spatial memories. However, this does not seem to be an exclusive function of LC-derived hippocampal DA. It was shown that activation of release from DA fibers from the VTA/SNc in the dorsal hippocampus of mice during spatial acquisition of a crossword maze also enhances memory retention by stabilizing new hippocampal maps and memory of new goal locations (McNamara et al., 2014). In rats, optogenetic stimulation of VTA/SNc fibers in dCA1 enhanced place cell activity, inhibited hippocampal interneurons, and shifted the center of mass of place fields (Mamad et al., 2017). Furthermore, a study in rats demonstrated that enhancing DA release in the dCA1 by optogenetic activation of VTA/SNc fibers after training in a conditioned-place-aversion task facilitates the persistence of long-term memory (Kramar et al., 2021). Thus, it is evident that DA contributions from both sources (i.e., VTA/SNc and LC) have the ability to modulate specific aspects of spatial memory formation in rodents.

Recently, it was shown that DA originating from VTA/SNc can also modulate associative memory formation (Tsetsenis et al., 2021). Specifically, photostimulation of dCA1 dopaminergic axons during contextual fear acquisition improves memory formation and recall 24 h later. Moreover, by genetically ablating all catecholamine contributions from the LC the same study demonstrated that DA originating from VTA/SNc is sufficient to support normal associative memory formation in the dorsal hippocampus during cFC. This notion finds further support from studies using a mouse model of early-stage Alzheimer’s disease (Nobili et al., 2017). These mice are characterized by neurodegeneration specifically in VTA dopaminergic neurons, and the neurodegeneration does not affect the LC cell population. It was shown that dopaminergic cell death in the VTA of these mice correlates with impairments in CA1 memory performance in cFC.

Another recent study reported that DA from LC cells projecting to dCA1 does not affect contextual memory formation (Chowdhury et al., 2022), further supporting the importance of midbrain dopaminergic inputs (Tsetsenis et al., 2021). In contrast, this work showed that LC to dCA1 connections, *via* the release of DA, modulate contextual memory linking, which is a process where an aversive memory acquired in a specific context can be activated in a different linked context. These studies demonstrate how DA from either the midbrain or the LC can contribute to different aspects of associative memory in the dorsal hippocampus (Table 1).

**Table 1:** Summary table of circuit-specific studies showing the effects of different sources of DA on hippocampus-dependent processes.

**Paradigm**	**Species**	**DA Source**	**Methodology**	**Major Finding**	**Caveats**	**Reference**
Crossword Maze	Mice	VTA/SNc	ChR2 expression in VTA/SNc of DAT-Cre mice with terminal stimulation in dCA1.	Photostimulation of dCA1 dopaminergic axons during spatial learning stabilizes new hippocampal maps and memory of new goal locations.	No demonstration of DA release upon photostimulation.	McNamara et al. (2014)
Food-baited Open Field	Rats	VTA/SNc	ChR2 expression in VTA/SNc of TH-Cre rats with terminal stimulation in dCA1.	Optogenetic stimulation of VTA/SNc fibers in dCA1 enhanced the activity, and shifted the center of mass of place fields.	No demonstration of DA release upon photostimulation.	Mamad et al. (2017)
Event arena	Mice	LC	ChR2 expression and light stimulation in LC or VTA of TH-Cre mice with pharmacological manipulations in dCA1.	Optogenetic activation of LC-TH+ neurons enhances memory persistence *via* dopamine D1/D5 receptors.	No demonstration of DA release from NE terminals. LC photostimulation could engage the VTA. Different methods were chosen to silence VTA vs. LC.	Takeuchi et al. (2016)
Spatial object recognition, Barnes Maze	Mice	LC	ChR2 expression in LC of TH-Cre mice with terminal stimulation and pharmacological manipulations in dCA1.	Dopamine release from the LC to the dorsal hippocampus improved spatial object recognition *via* dopamine D1/D5 receptors. Optogenetic stimulation of LC terminals in the dorsal hippocampus during training also improved spatial memory in the Barnes Maze task.	The Barnes maze experiments did not include pharmacological manipulations to shown if they are mediated by DA.	Kempadoo et al. (2016)
Conditioned Place Aversion	Rats	VTA/SNc	ChR2 expression in VTA/SNc of DAT-Cre rats with terminal stimulation in dCA1.	Stimulation of VTA DA fibers in dCA1 enhances memory persistence of CPA.	No demonstration of DA release upon photostimulation.	Kramar et al. (2021)
Contextual Fear Conditioning	Mice	VTA/SNc	ChR2 expression in VTA/SNc of DAT-Cre mice with terminal stimulation in dCA1.	Photostimulation of dCA1 dopaminergic axons during encoding improves contextual memory recall.	No demonstration of DA release upon photostimulation.	Tsetsenis et al. (2021)
Contextual Fear Conditioning	Mice	LC	Expression of an inhibitory DREADD in LC neurons that project to the hippocampus.	Chemogenetic inhibition of LC to dCA1 projecting neurons impairs contextual memory linking, without affecting contextual memory formation. This impairment is rescued by D1 receptor activation.	No direct evidence that the effects on memory linking are due to DA release from LC neurons projecting to the dCA1.	Chowdhury et al. (2022)

A general caveat of most of these studies is the inability to detect increases in DA upon stimulation, due to the lack of sensititve DA sensors. CPA, conditioned place aversion; DREADD, designer receptor exclusively activated by designer drugs.

## Conclusions

The experimental evidence reviewed here strongly supports an important modulatory role for DA in the regulation of long-term plasticity in the hippocampus with direct effects on learning and memory. This neuromodulatory action is largely mediated by activation of D1-type receptors in the dorsal hippocampus, which facilitates LTP and promotes spatial and contextual information encoding and storage (Hansen and Manahan-Vaughan, 2012). A possible mechanism is that DA signaling in the hippocampus lowers the threshold for synaptic plasticity that underlies learning and broadens the timing window between presynaptic and postsynaptic activity (Yang and Dani, 2014). Another possibility is that DA acts as a regulator of different inputs onto dCA1 pyramidal cells (Rosen et al., 2015). These cells receive and integrate information from the CA3 *via* the Schaffer collateral (SC) pathway and directly from the entorhinal cortex *via* the temporoammonic (TA) pathway. DA may act to rebalance the relative weights of SC and TA inputs by modulating the intrinsic and synaptic properties of hippocampal GABAergic interneurons, in a similar manner to the effects recently shown for acetylcholine (Palacios-Filardo et al., 2021).

Another level of complexity in deciphering the function of hippocampal DA in regulating cognitive processes is added when one considers the origin of the dopaminergic signal. Recent evidence from studies using optogenetic methods to enhance DA release in the dorsal hippocampus showed that both the VTA/SNc and the LC can release DA in the dCA1 to facilitate specialized aspects of learning (Table 1). How these two signals that act primarily through the same mechanism of D1-type receptor activation can produce diverse behavioral outcomes is still not completely clear. It is possible that VTA/SNc dopaminergic fibers target different synapses or cell populations in the dorsal hippocampus allowing for different types of neuromodulatory action (McNamara and Dupret, 2017). While innervation from the midbrain may be sparser, it can be equally efficient in releasing DA since the extracellular concentration of DA released from LC fibers constitutes only a small fraction of the amount of NE released (Kempadoo et al., 2016). In fact, the amount of NE co-released with DA from LC fibers should not be disregarded as NE can also influence associative memory formation in the dCA1 (Tsetsenis et al., 2022). Moreover, NE has a high affinity and has been shown to act as a potent agonist for D2-type receptors (Lanau et al., 1997; Sanchez-Soto et al., 2016), suggesting that it can activate dopaminergic signaling pathways independently from DA. Also, experiments that rely on LC soma activation should be designed with caution, as it is possible that the observed effects mediated by dopamine receptors in the hippocampus may be due to DA release from VTA/SNc terminals, *via* activation of LC-VTA inputs (Simon et al., 1979; Mejias-Aponte et al., 2009; Shelkar et al., 2017) and not necessarily attributed to release from noradrenergic terminals. Future research aiming to dissociate the functionality of DA release from these loci would benefit from the development of sensitive techniques for detecting DA in the hippocampal formation as well as discriminating DA from NE release (Dong et al., 2022).

## Author contributions

TT and JB wrote the initial draft of the manuscript. JD edited and wrote the final manuscript and contributed with financial support. All authors contributed to the article and approved the submitted version.
